# State anxiety by itself does not change political attitudes: A threat of shock experiment

**DOI:** 10.3389/fpsyg.2022.1006757

**Published:** 2022-12-01

**Authors:** Ulrich W. D. Müller, Oke Bahnsen, Georg W. Alpers

**Affiliations:** ^1^Department of Psychology, School of Social Sciences, University of Mannheim, Mannheim, Germany; ^2^Department of Political Science, School of Social Sciences, University of Mannheim, Mannheim, Germany

**Keywords:** anxiety, political attitudes, political ideology, threat of shock, implicit attitudes, pre-registered experiment

## Abstract

Previous research suggests that state anxiety may sway political attitudes. However, previous experimental procedures induced anxiety using political contexts (e.g., social or economic threat). In a pre-registered laboratory experiment, we set out to examine if anxiety that is unrelated to political contexts can influence political attitudes. We induced anxiety with a threat of shock paradigm, void of any political connotation. All participants were instructed that they might receive an electric stimulus during specified threat periods and none during safety periods. Participants were randomly assigned to one of two conditions: Political attitudes (implicit and explicit) were assessed under safety in one condition and under threat in the other. Psychometric, as well as physiological data (skin conductance, heart rate), confirmed that anxiety was induced successfully. However, this emotional state did not alter political attitudes. In a Bayesian analytical approach, we confirmed the absence of an effect. Our results suggest that state anxiety by itself does not sway political attitudes. Previously observed effects that were attributed to anxiety may be conditional on a political context of threat.

## Introduction

The role of anxiety in politics has attracted growing interest among the public. Rising anxiety levels are often seen as fueling right-wing populism, and this mechanism is considered a potential explanation for the rise of right-wing politicians and parties (e.g., Lussenhop, 2016). Discussions in the media suggest that anxiety sways voters to adopt more right-wing or conservative^1^ attitudes. In the political realm, triggers for this emotion can be manifold: for example, rising immigration, alienating globalization, threatening terrorism. Some politicians on the right political spectrum appear to evoke anxiety in their political campaigns intentionally; former US president, Donald Trump, has often been accused of it.

“Trump stoked the collective anxieties of millions of Americans by fixating on presumed threats: Islam. Globalization. Rapacious bankers. War-happy neocons. Trade deficits. Scheming reporters” (Caryl, 2018). However, there are similar examples from the left political spectrum. For example, in 1964, Lyndon B. Johnson’s campaign advertisement famously depicted a nuclear explosion implying that Barry Goldwater dealt carelessly with nuclear weapons (Jamieson, 1996, Chapter 5). Thus, it remains unclear whether it is a strategy to sway voters to the right or to intensify other existing attitudes as well.

Similarly, the scholarly literature has generated two conflicting expectations about the nature of the relationship between anxiety and political attitudes: the conservative shift and the ideological intensification hypothesis (for a review in the context of terrorist threat, see Huddy and Feldman, 2011). According to the conservative shift hypothesis, anxiety makes individuals adopt more conservative views. The conservative shift hypothesis is based on the theory of Motivated Social Cognition (Jost et al., 2003). Interestingly, this theory does not specify that the effect of state anxiety on political attitudes is limited to threat that occurs in a political context. This model postulates that the (chronic or temporary) psychological need to manage threat and uncertainty evoked by environmental influences can lead to an endorsement of core aspects of political conservatism, i.e., attachment to the status quo and hierarchy. The model states that individual dispositions such as self-interest (e.g., Sears and Funk, 1991) and personality (e.g., Jonason, 2014) can lead to an endorsement of conservatism. Moreover, it also postulates that situational influences can trigger state anxiety, which refers to the temporary anxiety level based on the momentary situation. State anxiety in turn is suggested to promote political conservatism.

On the other hand, the ideological intensification hypothesis states that anxiety polarizes existing political attitudes (Greenberg and Jonas, 2003). Accordingly, conservatives would become more conservative and liberals more liberal. While this hypothesis is at its core also based on the theory of motivated social cognition, the authors take issue with some facets of the original model. First, they object that the described core components of political conservatism, namely attachment to the status quo and hierarchy, are uniquely associated with political conservatism. Instead, they argue that these components can be identified on both sides of the political spectrum, in far-right as well as far-left political positions. Therefore, endorsement of either political view could serve to manage threats and uncertainty. Sometimes the ideological intensification hypothesis is also derived from terror management theory, which contends that threatening situations lead individuals to strengthen their established cultural worldviews (Anson et al., 2009; Castano et al., 2011).

Several empirical investigations have been reported in support of both, the conservative shift (e.g., Thórisdóttir and Jost, 2011) and the ideological intensification hypothesis (e.g., McGregor et al., 1998). Meta-analyses (Jost et al., 2003, 2017; Burke et al., 2013; Onraet et al., 2013) conclude that evidence favors the perspective that anxiety leads to a conservative shift.

However, it is still questionable whether the existing studies can causally identify the effect of anxiety: Even in experimental work anxiety inductions are typically loaded with potentially confounding semantic context of political opinion (see also Hatemi and McDermott, 2020). For example, previous studies induced anxiety using social or economic threat as triggers (e.g., Brader et al., 2008; Thórisdóttir and Jost, 2011). Typically, these are causally related to political issues, e.g., showing a video of the burning World Trade Center (Albertson and Gadarian, 2015, Chapter 5). Such manipulations do not only evoke anxiety, but instead, they can directly affect political attitudes. For example, priming terrorism affects attitudes toward national security policy that are not anxiety-related (see Huddy and Feldman, 2011). Also, mortality salience experiments, in which death anxiety is elicited by reminding individuals of their mortality (Burke et al., 2010, 2013), entail social triggers that may have unintended concomitant consequences. These mortality primes do not only evoke anxiety but all sorts of potentially confounding effects, e.g., a sense of belonging to others in the direct social surroundings (e.g., Jost et al., 2017, p. 331). Moreover, politically relevant topics are often directly or indirectly associated with death, e.g., terrorism, abortion, health care, and capital punishment (Burke et al., 2013). Taken together, it is unclear if the change in political attitudes detected in existing studies is due to anxiety or unintended consequences of the manipulation.

To the best of our knowledge, only one study on anxiety and politics intended to use an anxiety induction that is not intrinsically linked to political attitudes (Renshon et al., 2015). A group that had watched a threatening video clip showed more anti-immigration attitudes than a control group who watched a neutral clip. However, in the clip, the hero attempts to rescue a female mountain climber dangling over a precipice, which is clearly associated with social values as much as the dependent variable, i.e., attitudes toward immigrants. This may not capture political ideology at large.

Beyond such experimental studies converging evidence for the anxiety-conservatism link comes from research on the association between trait anxiety and political attitudes. Trait anxiety, in contrast to state anxiety, is a relatively stable predisposition to experience anxiety across various situations. There is an ongoing debate about a potential link between physiological sensitivity to threat and political attitudes. While some studies found a positive association between threat sensitivity and conservative attitudes (Oxley et al., 2008; Mustafaj et al., 2022), others did not (Bakker et al., 2020; Osmundsen et al., 2022). An association between threat sensitivity and conservative attitudes has been attributed to altered amygdala function found in conservative individuals (Pedersen et al., 2018). Although lower threat sensitivity is intrinsically linked to higher anxiety levels, this research can only be seen as supplemental evidence for a possible relation between state anxiety and political attitudes. First, threat sensitivity is an individual predisposition and is thus directly related to trait anxiety rather than state anxiety. Second, this work is based on observations rather than experimental evidence limiting its explanatory value for causality.

We fill this gap in existing research on the nexus between state anxiety and political attitudes by conducting a laboratory experiment using an established anxiety induction, often used in psychological research to model anxiety in the laboratory (Bublatzky et al., 2018). The threat of shock paradigm is well established in research on anxiety (Grillon et al., 1991) and is clearly free of any content remotely associated with politics. In such an experiment, participants are instructed that they might receive an electric shock during signaled threat periods and no shocks during safety periods (e.g., Drabant et al., 2011; Bublatzky et al., 2014). The screen’s background color indicates alternating periods of threat and safety. In our design, participants were randomly assigned to one of two experimental conditions where diverse measures of political attitudes were assessed under threat or safety. As a manipulation check, we measured relevant physiological indices of anxious arousal (skin conductance and heart rate).

In order to also tackle a potential limitation of previous experimental research, we assessed diverse measures of political attitudes. Previous work measured attitudinal shifts almost exclusively explicitly, which is prone to be biased by social desirability (Berinsky, 2004; Brownback and Novotny, 2018). Because social desirability is related to reporting more left-leaning political attitudes (Verhulst et al., 2010), this may be particularly problematic. Thus, we tested the effects of the anxiety induction not only on explicit but also on implicit measures of political attitudes.

## Materials and methods

### Participants

75 individuals participated in the laboratory experiment (38 in Condition 1 and 37 in Condition 2). Before running the experiment, we conducted power calculations. We set α = 0.05 and β = 0.2. Previous mortality salience manipulations found medium to large effects on conservative political attitudes (e.g., Jost et al., 2004, effect of size *d* = 0.6). Therefore, our power calculations were conservative with a Cohen’s *d* = 0.4, suggesting a sample size of about *N* = 100.

However, the COVID-19 pandemic forced us to terminate data collection after *N* = 75 participants. A sequential blocking procedure based on data from a screening determined their random assignment to one of the two experimental conditions. To account for the smaller sample size, we calculated Bayes factors in the case of null results.

Participants assigned to the conditions did not differ significantly in age, sex, education, or social and economic conservatism scores (see Appendix 1.1). Overall, the mean age was 30.93 years (range: 18–63). There were 42 women and 33 men.

### Material and apparatus

As a manipulation check, participants rated the hedonic valence and arousal of the threat and safety cue using the Self-Assessment Manikin (SAM; Bradley and Lang, 1994). Moreover, participants rated the perceived threat using a visual analog 10-point scale ranging from not at all (1) to highly threatening (10).

As a conventional explicit measure of political attitudes, we used an 11-point Left–Right Self-Placement scale. This scale was presented as visual analog scale, i.e., as a horizontal line that represented a continuous scale; the left of this line is labeled by “Very Left” and the right by “Very Right.” By clicking on the line, participants selected a position along this continuous scale which was later scored as 0 (very left) to 10 (very right).

Furthermore, as an explicit multidimensional measure of political attitudes with social and economic dimensions, we administered the Social and Economic Conservatism Scale (SECS; Everett, 2013). This measure consists of 12 words representing issues important to social or economic conservatism (7 related to social conservatism and 5 to economic conservatism). On a feeling thermometer, individuals report how positive or negative they felt regarding those words on a scale ranging from “Very Negative” to “Very Positive” on a visual analog scale (see Appendix 1.2). Values were later translated to 101 discrete values.

As an implicit measure of political attitudes, we used the Single-Target Implicit Association Test (ST-IAT), which can reliably and validly measure political attitudes (see Nosek et al., 2007; Bluemke and Friese, 2008). The ST-IAT assesses spontaneous evaluations and an individual’s association between a target concept and the attribute concepts “Positive” and “Negative.” Automatic evaluations are inferred from response times to a discrimination task (see section Procedure). Stimuli for the target concept “Right” and the attribute concepts “Positive” and “Negative” were taken from Bluemke and Friese (2008). For example, for the attribute concept “Positive,” we use the stimuli “Joy,” “Present,” “Love,” “Health,” and “Laughter” and for target concept “Right,” the stimuli “National Flag” and “Tradition.” The words for all other concepts can be found in Appendix 1.3. For the target concepts “Economic Conservatism” and “Social Conservatism,” we use the individual items of the SECS. Hence, we chose predominantly target concepts corresponding with right-leaning political attitudes. That is, we draw on the common one-dimensional bipolar conceptualization of conservatism and liberalism (see Jost et al., 2009, p. 312–313), meaning that more positivity toward right-leaning political attitudes is tantamount to less positivity toward left-leaning political attitudes and vice versa. To measure trait anxiety, we administered the Spielberger State–Trait Anxiety questionnaire’s trait scale, consisting of 20 items (STAI-T German version: Laux et al., 1981).

The experiment was programmed in OpenSesame (Mathôt et al., 2012). To induce anxiety, we used a well-established threat of shock paradigm consisting of signaled threat and safety periods (see Bublatzky et al., 2017). Electric pulses were generated by a constant current stimulator (DS7AH, Digitimer, Hertfordshire, United Kingdom). We used a vAmp amplifier (BrainProducts, Munich, Germany) to record psychological data.

### Procedure

The procedure was reviewed and approved by the ethics committee of the University of Mannheim (EK Mannheim, 11/2019). Moreover, we pre-registered the study (Müller et al., 2019). We recruited participants who were healthy and of age *via* local news outlets (print and online) and flyers. Eligible participants were invited to two sessions at least 1 day apart (a) the screening and (b) the actual participation in the laboratory study. In the screening procedure, participants reported several socio-economic characteristics (age, sex, and school education). In addition, participants placed themselves on an 11-point left–right scale. In addition, they answered two items of the Social and Economic Conservatism Scale (SECS: Everett, 2013), one item of the economic conservatism subscale (item “Welfare Benefits”), and one of the social conservatism subscale of the SECS (item “Traditional Values”). With this, we measured their political attitudes prior to the manipulation. We chose visual analog scales for the explicit measures of political attitudes to account for the fact that these measures were later on also taken in the laboratory study and respondents may remember their answers. All eligible participants were invited to the experiment in the lab. Left-leaning individuals volunteered more frequently. Moreover, we offered a monetary reimbursement for their time (7 € for approximately 45 min).

Data from the screening was used for sequential blocking to randomize assignment to conditions (Pocock and Simon, 1975; Jin et al., 2021). This guaranteed an approximately equal distribution of pre-existing political attitudes and relevant socio-economic characteristics in the two conditions. The experimenter was blind to the results of the screening.

In the laboratory, all participants gave written informed consent. Participants then underwent a brief shock workup to calibrate the intensity of the shocks to individual pain thresholds. For this, an electrode was attached to their upper arm, and shocks with increasing intensities were delivered (1 mA up to a maximum of 25 mA). Shocks consisted of three consecutive pulses with a duration of 2 and 250 ms intervals. Participants were instructed to evaluate the sensation after each shock until it was maximally unpleasant but not yet painful (Drabant et al., 2011). Participants were told that the final intensity of the shock-workup will be used for the electric stimulus during the experiment. After this, no more shocks were delivered (see also Bublatzky et al., 2010, 2014). Next, additional electrodes were attached to assess skin conductance and heart rate as physiological indices of anxious arousal.

Then, participants were told that they could get an electric shock during specific periods, signaled by a particular screen color (threat phase). In contrast, no shocks would be delivered during other periods with another screen color (safety phase). Colors (e.g., yellow for threat and red as safe – or vice versa) were counterbalanced.

**Figure 1:**
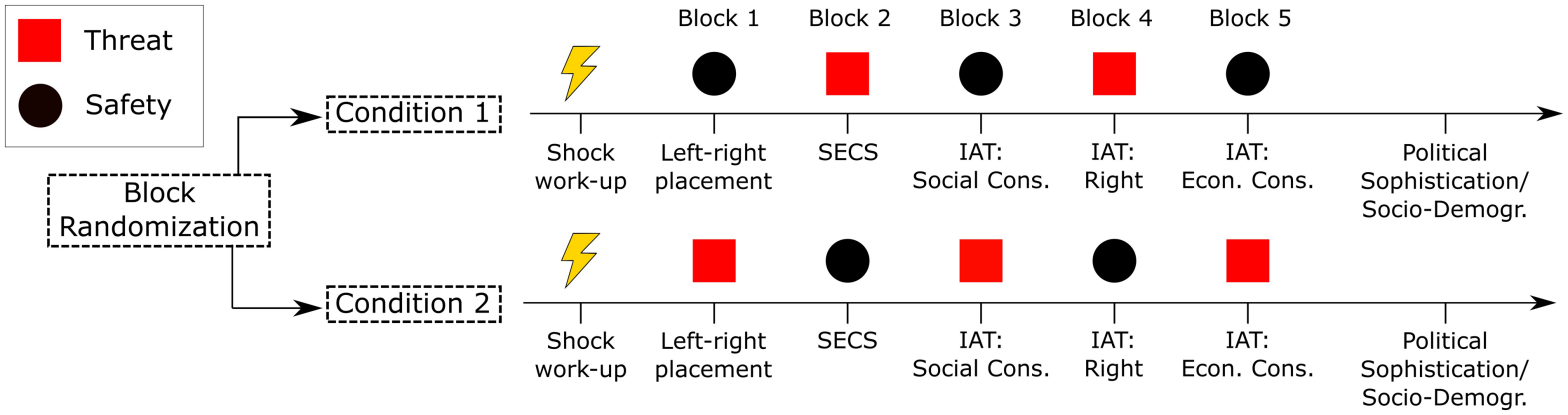
Experimental design. SECS, Social and Economic Conservatism Scale; IAT, Single-Target Implicit Association Task.

All participants went through five blocks of alternating safety and threat periods per block (see Figure 1). To compare various measures taken under threat and safety, half of the participants started Block 1 under safety (Condition 1) and the other half under threat (Condition 2). Because the threat of shock may not be sustained over a long time period, alternation between threat and safety is an essential feature of the paradigm.

At the beginning of each block, we measured physiological indicators as a 5-s baseline period. Participants were instructed to look at the blank screen attentively during these periods. This was followed by a 10-s measurement period during which the color cue of the corresponding block (yellow or red) was shown. In addition, before Block 1 started, participants rated valence, arousal, and perceived threat of the threat and safety cue.

In Block 1, participants reported their Left–Right Self-Placement. In Block 2, we assessed the items of the SECS (Everett, 2013). Next, participants completed a practice trial for the IATs, followed by the three IAT blocks (Blocks 3 to 5) measuring implicit attitudes regarding the target concepts “Social Conservatism,” “Right,” “Economic Conservatism,” consecutively.

Table 1 provides an overview of the procedure in the IAT blocks. In the beginning of Block 3, as an initial practice trial, 10 words (5 positive and 5 negative) of the attribute concepts “Negative” or “Positive” were successively shown in random order, where each word was presented twice. The left response key was assigned to the positive words and the right response key to the negative words for the practice exercise. Participants were instructed to click on the corresponding response key as fast as possible after a word was displayed. Subsequently, Block 3 to Block 5 each consisted of two parts. The first part was similar to the practice exercise, but now also words related to the target concept were either assigned to the same key as the positive or negative words. In the second part, the key assignment for the target concept was inverted, mapping the target concept on the same key as the other attribute concept. The key assignment for the attribute concepts alternated between blocks. In each part, 5 positive words, 5 negative words, and the words referring to the target concept were shown in random order, and each word was displayed twice.

**Table 1:** Concept assignment, stimulus, and trial proportions across ST-IAT parts.

Part of IAT	Block	Left key concept	Right key concept	Number of stimuli (trials)		
				Positive	Negative	Target concept
Practice	3	Positive	Negative	5 (10)	5 (10)	-
1-Sc. Cons.	3	Negative + Sc. Cons.	Positive	5 (10)	5 (10)	6 (12)
2-Sc. Cons.	3	Negative	Positive + Sc. Cons.	5 (10)	5 (10)	6 (12)
1-Right	4	Positive + Right	Negative	5 (10)	5 (10)	7 (14)
2-Right	4	Positive	Negative + Right	5 (10)	5 (10)	7 (14)
1-Ec. Cons	5	Positive + Ec. Cons.	Negative	5 (10)	5 (10)	4 (8)
2-Ec. Cons	5	Positive	Negative + Ec. Cons.	5 (10)	5 (10)	4 (8)

Sc. Cons, Social conservatism; Ec. Cons, Economic conservatism.

After the experiment, participants indicated whether the threat/safety instructions were perceived as convincing (yes/no) and whether they affected them (yes/no). Moreover, participants filled in the STAI-T (Laux et al., 1981) and answered three questions assessing their political knowledge (whether the federal budget of Germany was balanced in 2018, how high the electoral threshold for German federal elections is, and whether the primary or secondary vote is decisive German federal elections). In addition, we assessed occupational education, party identification, and household income (see Roßteutscher et al., 2018). Finally, participants were debriefed and invited to confirm consent to use their data.

### Data recording and preparation

Skin conductance responses (SCRs) were measured by two Ag/AgCl electrodes attached to the palm of the participants’ non-dominant hand (constant voltage of 0.5 V; 500 Hz sampling rate). Data was processed in BrainVision Analyser 2.2 (BrainProducts, Munich, Germany) according to guidelines (Boucsein, 2012). Specifically, data was pre-processed using a 2 Hz FIR low-and a 0.05 Hz high-pass filter. Peak SCRs during the 10-s measurement bins were baseline-corrected with respect to 1 s before the onset of the color and extracted per block for every participant. Data were normalized to T-scores.

Heart rate responses were assessed with a sampling rate of 1,000 Hz, and frequencies below 0.1 and above 12 Hz were filtered. Detection of R-wave, visual inspection, and calculation of heart rate in bpm were executed in Brain Vision Analyser 2.2 (BrainProducts, Munich, Germany). Heart rate averages for the measurements bins per second were baseline-corrected regarding the 5-s before the onset of the color and extracted per block for every participant.

For calculating IAT scores (Greenwald et al., 2003) we skipped wrong responses and recoded very fast and very slow responses according to Bluemke and Friese (2008). Individual differences in reaction times were controlled for by z-transformations using the individual means and the individual standard deviations (see Bluemke and Friese, 2008). We calculated IAT scores by subtracting mean reaction times in the “Positive + Target Concept” blocks from the mean reaction times in the “Negative + Target Concept” blocks for each participant (see Bluemke and Friese, 2008). A positive value implies that a participant associates the target concept more strongly with positive than negative stimuli, i.e., an implicit preference in favor of the target concept.

Subgroups of left-leaning and right-leaning participants for the different analyses were created based on their responses in the screening (see Appendix 1.4 for an overview of the screening responses). For the analyses of the Left–Right Self-Placement and the IAT for the concept “Right,” subgroups were based on the participants’ responses to the Left–Right Self-Placement in the screening (left-leaning subgroup: Left–Right Self-Placement of less than 5, right-leaning subgroup: Left–Right Self-Placement of greater than 5). Subgroups for the SECS and the IAT SECS analyses were based on the mean of the responses on the two SECS items that we assessed during the screening, while for the subscales (Social Conservatism, Economic Conservatism) they were only based on the item of corresponding that scale (left-leaning subgroup: score of <50; right-leaning subgroup: score of >50).

## Results

### Manipulation check

#### Explicit measures

**Figure 2:**
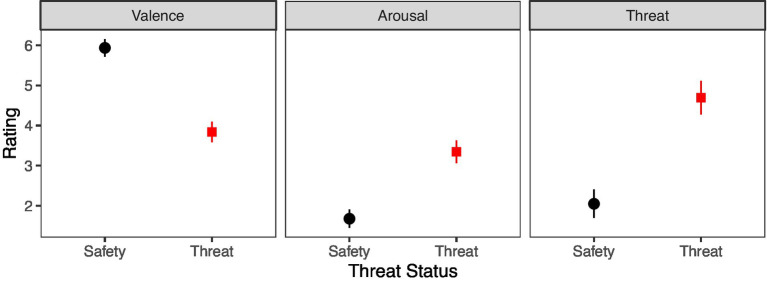
Mean ratings of valence, arousal, and threat for the assigned safety and threat colors. Bars indicate 90% confidence intervals.

Most participants found the threat/safety instructions convincing (70 of 75) and felt affected by the threat/safety instructions (56 of 75). Valence, arousal, and threat ratings of the participants of the safety and threat colors further confirmed the successful induction of anxiety (Figure 2). Participants rated the respective safety color significantly more positive (*M* = 4.16, *SD* = 1.75) than the threat color (*M* = 2.07, *SD* = 1.50), *t*(74) = 8.31, *p* < 0.001. Moreover, participants rated the safety color significantly less arousing (*M* = 4.65, *SD* = 1.94) than the threat color (*M* = 6.32, *SD* = 1.57), *t*(74) = 6.98, *p* < 0.001. Furthermore, participants rated the safety color significantly less threatening (*M* = 2.05, *SD* = 2.41) than the threat color (*M* = 4.69, *SD* = 2.85), *t*(74) = 6.91, *p* < 0.001.

Overall, most participants rated the manipulation credible and effective, and explicit measures confirmed that anxiety induction was successful.

#### Skin conductance responses to threat and safety

**Figure 3:**
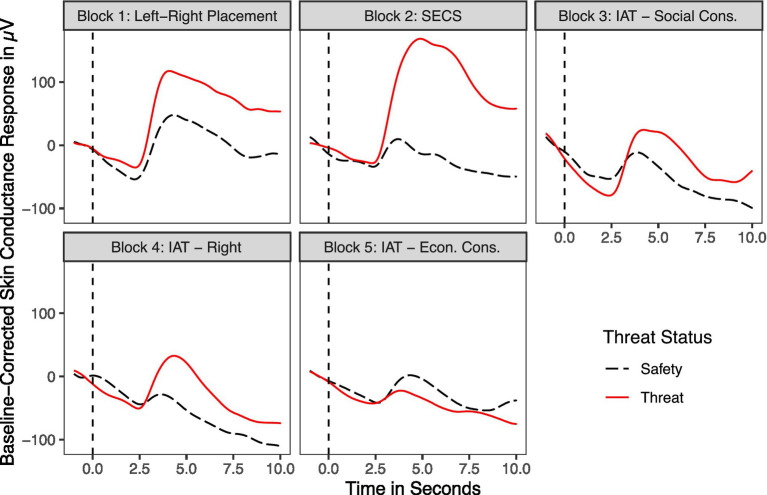
Mean baseline-corrected skin conductance levels per block for 10s after the onset of the threat and saftey phase. Averages are shown separately for experimental conditions. Participants under threat are indicated by red lines and participants under safety by black lines.

The analysis of skin conductance further demonstrated that anxiety induction was successful (Figure 3). Peak skin conductance responses of participants that completed Block 1 under threat (*M* = 52.68, *SD* = 11.77) were significantly higher than the peak-skin conductance responses of those that completed it under safety (*M* = 47.39, *SD* = 7.15), *t*(73) = 2.34, *p* = 0.023. This was also the case in Block 2, as the peak-skin conductance responses of participants that completed Block 2 under threat (*M* = 53.64, *SD* = 11.95) were significantly higher than the peak-skin conductance level of those that completed it under safety (*M* = 46.26, *SD* = 5.51), *t*(73) = 3.45, *p* = 0.001. Descriptively, this expected difference persisted in Blocks 3 and 4 (not in Block 5) but missed significance, (Block 3: *t*(73) = 1.48, *p* = 0.145; Block 4: *t*(73) = 0.60, *p* = 0.554; Block 5: *t*(73) = 0.92, *p* = 0.362).

#### Heart rate responses to threat and safety

**Figure 4:**
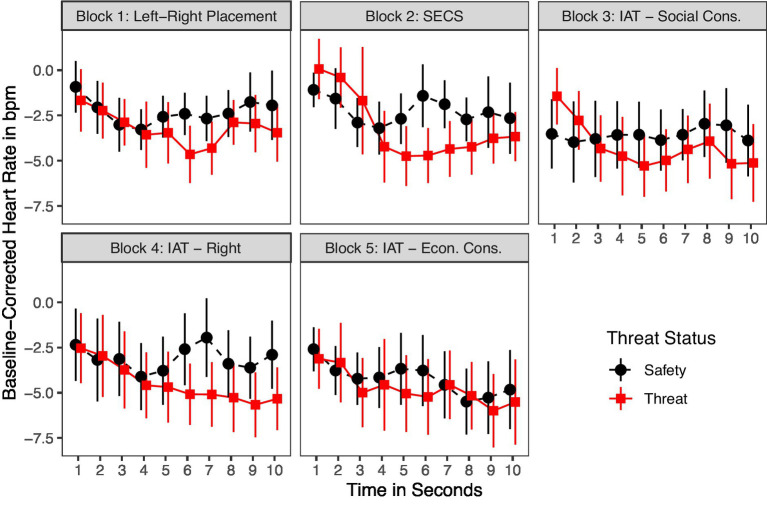
Mean baseline-corrected heart rate response per block for 10s after the onset of the threat and saftey phase. Averages are shown separately for experimental conditions. Participants under threat are indicated by red lines and participants under safety by black lines. In addition, 90% confidence intervals are displayed.

Heart rate responses also confirmed the success of our anxiety induction (Figure 4). We found the most profound differences 6 s after the onset of the threat and safety phase (see also Appendix 1.5 for visualizations of statistical tests). Therefore, we tested whether these differences are statistically significant. In Block 1, the average baseline-corrected heart rate response under threat was lower (*M* = −4.65, *SD* = 4.27) than under safety (*M* = −2.41, *SD* = 5.72), although this difference was only significant on the 90% level of confidence, *t*(73) = 1.92, *p* = 0.06. In Block 2, heart rate responses under threat (*M* = −1.41, *SD* = 5.55) were significantly lower on the 95% level of confidence than under safety (*M* = −4.71, *SD* = 6.26), *t*(73) = 2.42, *p* = 0.018. This effect persisted descriptively in the later blocks but missed significance [Block 3: *t*(73) = 0.78, *p* = 0.435; Block 4: *t*(73) = 1.61, *p* = 0.113; Block 5: *t*(73) = 0.86, *p* = 0.394]. This more pronounced deceleration in the threat than the safety phase indicates a successful manipulation (Bradley et al., 2005; Bublatzky et al., 2017).

### Testing the conservative shift hypothesis

First, we tested the conservative shift hypothesis. For the explicit and implicit measures, there was no statistically significant differences between participants assessed under threat and those assessed under safety. The results of *t*-tests show that the participants assessed under threat did not score differently than participants assessed under safety on Left–Right Self-Placement, *t*(73) = 0.31, *p* = 0.757, SECS score, *t*(73) = 0.87, *p* = 0.389, the Social Conservatism Subscale of the SECS, *t*(73) = 1.06, *p* = 0.293, or the Economic Conservatism Subscale of the SECS, *t*(73) = 1.06, *p* = 0.931. Also, the participants assessed under threat did not score differently than participants assessed under safety on the IAT measures for the concepts “Right,” *t*(73) = 0.17, *p* = 0.867, “Social and Economic Conservatism,” *t*(73) = 0.99, *p* = 0.327, “Social Conservatism,” *t*(73) = 1.02, *p* = 0.313, or “Economic Conservatism,” *t*(73) = 0.11, *p* = 0.911. In sum, there is no evidence for the conservative shift hypothesis.

**Figure 5:**
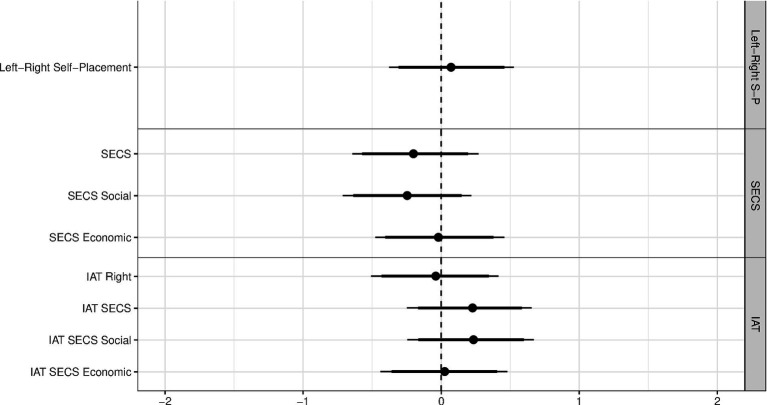
Effect of threat of shock on explicit and implicit political attitudes as Cohen’s *d* effect sizes. Positive values imply that average political attitudes under threat are more conservative than those under safety. Thin (thick) bars show the 95% (90%) bootstrap BCa confidence intervals. The pooled standard deviation was used for calculating Cohen’s *d*. IAT, Implicit Association Task; SECS, Social and Economic Conservatism Scale. *N* = 75.

We calculated Cohen’s *d* (Cohen, 1988; see Figure 5). The absolute values of the point estimates for Cohen’s *d* were at maximum 0.25 (SECS Social Conservatism Subscale), which is conventionally considered a small effect, but might be meaningful in pre-registered studies, seen the generally considerable lower effect sizes found in pre-registered studies (Schäfer and Schwarz, 2019). More importantly, the effects showed no consistent pattern regarding their direction.

We explored further possible evidence for the conservative shift hypothesis. Specifically, we ran linear regressions of the outcome variables on experimental condition, prior political attitudes, age, and gender which did not turn up any effects (Appendix 1.6). Moreover, we also probed the SECS on an item level but did not find any effects either (Appendix 1.7).

### Testing the ideological intensification hypothesis

To test whether there is an effect of anxiety on political attitudes conditional on participants’ pre-existing political attitudes, we separately considered the groups of left- versus right-leaning participants (see Appendix 1.4). Regarding the explicit and implicit measures of political attitudes, we did not find significant differences between the participants assessed under threat and those assessed under safety, both for the left-leaning and the right-leaning subgroup. For the left-leaning subgroup, the participants assessed under threat did not score differently than participants assessed under safety on the Left–Right Self-Placement, *t*(59) = 0.15, *p* = 0.885, SECS score, *t*(44) = 0.60, *p* = 0.549, the Social Conservatism Subscale of the SECS, *t*(31) = 0.31, *p* = 0.763, the Economic Conservatism Subscale of the SECS, *t*(52) = 0.18, *p* = 0.857 as well as the IAT measures for the concepts “Right,” *t*(59) = 0.19, *p* = 0.847, “Social and Economic Conservatism,” *t*(44) = 0.17, *p* = 0.987, “Social Conservatism,” *t*(31) = 0.59, *p* = 0.558, and “Economic Conservatism,” *t*(52) = 0.02, *p* = 0.982. Also for the right-leaning subgroup, the participants assessed under threat did not score differently than participants assessed under safety on Left–Right Self-Placement, *t*(5) = 0.508, *p* = 0.639, the SECS score, *t*(25) = 0.46, *p* = 0.650, the Social Conservatism Subscale of the SECS, *t*(40) = 1.12, *p* = 0.270, the Economic Conservatism Subscale of the SECS, *t*(19) = 1.16, *p* = 0.261, as well as the IAT measures for the concepts “Right,” *t*(5) = 0.003, *p* = 0.998, “Social and Economic Conservatism,” *t*(25) = 1.79, *p* = 0.086, “Social Conservatism,” *t*(40) = 0.84, *p* = 0.405, and “Economic Conservatism,” *t*(19) = 0.36, *p* = 0.727.

**Figure 6:**
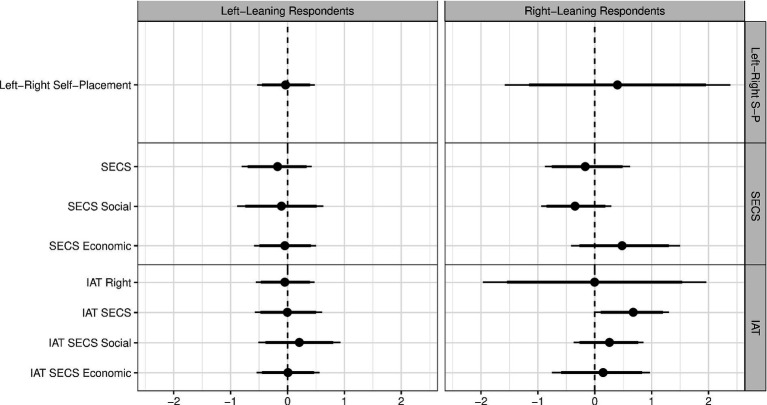
Effects of anxiety on measures of political attitudes in subgroups based on pre-existing political attitudes as Cohen‘s *d* effect sizes. Positive values imply that average political attitudes under threat are more conservative than those under safety. Thin (thick) bars show the 95% (90%) bootstrap BCa confidence intervals. The pooled standard deviation was used for the calculation. IAT, Implicit Association Task; SECS, Social and Economic Conservatism Scale. *N* varies per subgroup and measure; see also section Data recording and preparation (left panel: *N* = range: 31–61; right panel: *N* = range: 7–42).

To further investigate the robustness of these results, we calculated effect sizes (Cohen’s *d*, see Figure 6). The effects in the subgroups displayed no consistent pattern in terms of their direction, as would be expected under the ideological intensification hypothesis.

Again, we explored further analyses that consistently delivered no support for the validity of the ideological intensification hypothesis. Specifically, we ran separate linear regressions of the outcome variables on experimental condition, prior political attitudes, age, and gender (Appendix 1.8). Moreover, we investigated whether anxiety affected the scores of the SECS on the item level (Appendix 1.9).

As an additional robustness check (Appendix 1.10), we tested the ideological intensification hypothesis with more statistical power by considering participants on both the left and the right together in a pooled sample. We assessed for each scale whether the manipulation increased the absolute distance between an individual’s value and the scale midpoint. Because this is not the case, this corroborates that we cannot confirm the ideological intensification hypothesis.

#### Quantifying evidence for the null hypothesis using Bayes factors

We calculated Bayes factors to assess whether the *p*-values in the primary analysis are non-significant because effects are truly null or because of data insensitivity (Dienes, 2014). For the conservative shift hypothesis, the rival hypotheses are H_0_: δ = 0 and H_1_: δ > 0, while for the ideological intensification hypothesis they are H_0_: δ = 0 and H_1_: δ < 0 (H_1_: δ > 0) in the left-leaning (right-leaning) subgroup, where δ is defined as the effect size of the manipulation on our measures. As prior distribution for the alternative hypothesis, we selected a default option, namely a Cauchy distribution (Ly et al., 2016) with location of 0 and scale of $1/\sqrt{2}$. Because our alternative hypotheses are one-sided, we truncated this distribution at zero.

The Bayes factors for Bayesian independent samples *t*-test with the prior distribution defined above are shown in Table 2. Bayes factors in the direction of the null hypothesis (B_01_) quantify how much more likely the data are to be observed under the null hypothesis (H_0_) than under the alternative hypothesis (H_1_). By convention, the data provides substantive support for the null hypothesis if B_01_ is greater than three (Jeffreys, 1961). For the conservative shift hypothesis, the Bayes factors in the direction of the null hypothesis were greater than three for six out of the eight measures. These results provided substantive support for the null hypothesis relative to the conservative shift hypothesis. For the test of the ideological intensification hypothesis among the left-leaning participants, the Bayes factors in the direction of the null hypothesis were greater than three for six out of the eight measures. These results again provided substantive support for the null hypothesis relative to the ideological intensification hypothesis. The Bayes factors for the test of the ideological intensification hypothesis among the few right-leaning participants in our sample indicated, unsurprisingly, data insensitivity for six of the eight measures. Hence, the Bayes factors show that the left-leaning participants did not shift to the left under threat while the data did not provide evidence about whether the right-leaning participants shifted to the right under threat. Thus, summarizing, we found evidence for the absence of a conservative shift and an absence of an ideological intensification.

**Table 2:** Bayes factors in the direction of the null hypothesis for each measure and each hypothesis.

	Conservative shift hypothesis	Ideological intensification hypothesis	
	All participants	Left-leaning participants	Right-leaning participants
Left–right self-placement	3.28	3.44	1.31
SECS	7.16	2.10	3.63
SECS social	7.89	2.39	6.20
SECS economic	4.46	3.18	1.03
IAT right	4.72	3.31	1.84
IAT SECS	1.69	3.38	0.50
IAT SECS social	1.63	4.31	1.61
IAT SECS economic	3.85	3.70	2.00

## Discussion

Our data show that an anxious state by itself does not change political attitudes. In this study, we examined the effect of anxiety on explicit and implicit political attitudes, elicited by threat of shock, a well-established experimental anxiety induction. We used established explicit and implicit measures of political attitudes to assess its effect.

Although eliciting anxiety is often thought to play a central role in shaping political attitudes, existing experimental research on the nexus between state anxiety and political views did not examine it independent of contexts: Previously used anxiety manipulations potentially evoke not only anxiety but also other emotions and potentially affect political attitudes and behavior directly. Hence in contrast to our study, previous experimental research was not designed to test the different conflicting theoretical expectations, namely the conservative shift and the ideological intensification hypothesis (see Huddy and Feldman, 2011) in isolation of political contexts. Predominantly two types of experimental manipulations were used in previous work on the relationship between anxiety and political attitudes. In the first kind, anxiety was induced by presenting political or economic information or events directly related to political issues (e.g., Brader et al., 2008; Thórisdóttir and Jost, 2011). These manipulations may directly influence political attitudes independently of the elicited anxiety. Moreover, the manipulations do not necessarily evoke anxiety but rather a specific fear of a subject, object, or situation related to politics. This suggests that characteristics of the manipulations may be more relevant than the emotion in driving the effects on political attitudes found in previous studies. The second kind of design makes mortality salient to elicit death anxiety (Burke et al., 2010). This manipulation may also directly affect political attitudes independently of the elicited anxiety (see also Jost et al., 2017, p. 331).

Our study is the first to use a threat of shock anxiety induction to induce anxiety in this line of research. The threat of shock paradigm is a contemporary experimental model for anxiety, e.g., used to investigate the effect of anxiety on perceptual processes (Kavcıoğlu et al., 2021). This anxiety manipulation is free of political context. Moreover, implicit measurements of attitudes were rarely deployed in this specific area of research. Hence, in addition to the conventional explicit measures of political attitudes deployed in previous research, we utilized implicit measures of political attitudes, specifically, a Single Target Implicit Association Test (ST-IAT; Bluemke and Friese, 2008).

Our findings shed light on the potential relationship between state anxiety and political attitudes. The successful anxiety induction was confirmed by psychometric and physiological data (skin conductance responses and heart rate). However, in contrast to previous findings (Jost et al., 2003, 2017; Burke et al., 2013; Onraet et al., 2013), we did not find an effect of anxiety on explicit or implicit political attitudes. However, null results with respect to implicit attitudes might also be due to a no longer successful manipulation at the end of the experiment (as suggested by the manipulation checks). This specifically only concerned the blocks in which implicit attitudes were assessed. Moreover, the Bayesian analytical approach supports the absence of any effect of anxiety on political attitudes. This approach revealed that the non-significance of effects resulted from the circumstance that effects were truly null and not from data insensitivity (e.g., due to large standard errors because of small sample size).

Our findings contribute to the literature by showing that experimentally induced anxiety does not affect political attitudes, at least not by itself. Specifically, we neither find evidence for the conservative shift hypothesis (Jost et al., 2003) nor the ideological intensification hypothesis (Greenberg and Jonas, 2003). One explanation for the discrepancy with previous findings is that we elicited threat void of any political context, compared to previous studies that were mainly limited to inductions of political threat. This interpretation aligns with recent theoretical work that postulates that effects of threat on political attitudes should be conditional on specific threat contexts (Brandt and Bakker, 2022). More specifically political preferences that address the specific threat are fostered. For example, terrorism is thought to result in more support for political conservatism. Our investigation also speaks to the ongoing debate whether trait anxiety, in the form of (context unspecific) physiological sensitivity to threat, is related to political positions. While the influential study of Oxley et al. (2008) displayed that higher threat sensitivity correlates with more pronounced conservative attitudes, multiple replications objected to this claim (Bakker et al., 2020; Osmundsen et al., 2022). Although our investigation focused on state anxiety, threat sensitivity is in fact intrinsically linked to higher state anxiety in response to threat. Hence, our study underpins the findings of Bakker et al. (2020) and Osmundsen et al. (2022) because our results call a relationship between higher levels of state anxiety and more pronounced conservative political attitudes into question. Recent work suggests that threat sensitivity only predicts more specific attitudes like anti-immigrant attitudes (Mustafaj et al., 2022). However, we also did not find effects of threat on the immigration item of the SECS (see Appendix 1.7).

Some limitations of this study need to be acknowledged. First, while all explicit manipulation checks and the physiological indices during the earlier blocks clearly indicate that the manipulation was effective, we found that physiological arousal subsided in later blocks. Since these blocks contained the implicit measures of political attitudes, our findings regarding the effects on implicit political attitudes need to be interpreted with caution. However, a decrease in physiological arousal does not necessarily indicate that the manipulation was ineffective in the later blocks because habituation on physiological and subjective measures can be asynchronous (Lang and Craske, 2000; Müller et al., 2022). Second, due to the outbreak of the COVID-19 pandemic, our sample did not reach the planned size based on *ad hoc* power analysis. However, we confirmed the evidence for the null hypothesis by calculating Bayes factors. In addition, even the inconsistent directions of non-significant findings were incompatible with both the conservative shift and the ideological intensification hypothesis. Third, our sample is not representative of the (German) population. This raises the question of the generalizability of our findings, especially because most of the participants described themselves as left-leaning. Accordingly, we gained less insight into how anxiety affects the political attitudes of right-leaning individuals, considering their low number in our sample. Nonetheless, we were able to test both rival hypotheses solely by considering left-leaning individuals. This is because both hypotheses have different predictions about the effect on left-leaning people, which allowed us to test both hypotheses against each other.

Future research should replicate our findings in bigger samples. In addition, we recommend conceptual replication in a more representative sample, with more right-leaning participants. For this, other anxiety inductions unrelated to political attitudes could be used. In addition, individual differences associated with political attitudes should be assessed (e.g., Zmigrod and Goldenberg, 2021). Thereby, it could be investigated whether effects also not occur in specific subpopulations. This way, confidence in the interpretation of our findings, i.e., that anxiety by itself does not affect political attitudes can be further reinforced. Furthermore, systematic variations of contexts might shed light on the specific factors responsible for previously found effects of anxiety on political attitudes. The theoretical framework of affective intelligence theory (Marcus et al., 2011) may also be promising for future research. This theory is concerned with how fear alters information-seeking and, thus, may indirectly shape support for the far right (Marcus et al., 2019). Investigations in a context completely unrelated to politics would be interesting because existing empirical evidence is mainly derived from threat in political contexts, most prominently threat of terrorism (for a review see Godefroidt, 2022).

Taken together, our findings introduce a new perspective and methodological approach to the common notion that anxiety can influence political attitudes. Contrary to these previous beliefs, not the elicitation of anxiety *per se*, but the specific context in which anxiety is elicited is likely to make the difference. To borrow well-established paradigms from psychology in order to study political attitudes appears promising. This is the first study to demonstrate that anxiety void of political associations does not appear to alter political attitudes.

## Data availability statement

Data and Code are deposited at MADATA the data repository of the University of Mannheim and are available for future research: https://doi.org/10.7801/404.

## Ethics statement

The studies involving human participants were reviewed and approved by Ethics Committee of the University of Mannheim. The patients/participants provided their written informed consent to participate in this study.

## Author contributions

UM and OB engaged in the conceptualization, methodology, data curation, software programming, formal analysis, investigation, visualization, writing of the original draft, and review and editing of the manuscript. GA was involved in the conceptualization, methodology, review and editing of the manuscript, and provided resources and supervision. All authors contributed to the article and approved the submitted version.

## Funding

UM and OB were supported by the University of Mannheim’s Graduate School of Economic and Social Sciences (GESS), funded by the German Research Foundation (DFG), and the GESS Young Scholar Award. The publication of this article was funded by the Ministry of Science, Research and the Arts Baden-Württemberg and the University of Mannheim.

## Conflict of interest

The authors declare that the research was conducted in the absence of any commercial or financial relationships that could be construed as a potential conflict of interest.

## Publisher’s note

All claims expressed in this article are solely those of the authors and do not necessarily represent those of their affiliated organizations, or those of the publisher, the editors and the reviewers. Any product that may be evaluated in this article, or claim that may be made by its manufacturer, is not guaranteed or endorsed by the publisher.

## Footnotes

^1^We use the lables "right" and "conservative" ("left" and "liberal") synonymously.
