# Supplementary material for: State anxiety by itself does not change political attitudes: A threat of shock experiment
Source: Front Psychol. 2022 Dec 1;13:1006757. doi: 10.3389/fpsyg.2022.1006757 (PMC9752813; doi:10.3389/fpsyg.2022.1006757)
Supplement: Supplementary file 1 [file Data_Sheet_1.pdf]

Supplementary Material

1. Appendix

1.1. Randomization Check: Logit Model

	Dependent variable: Assignment to Condition 1	
	Coefficient	(Standard error)
Age	-0.006	(0.019)
Gender	0.557	(0.594)
Left-Right Placement (Pre)	0.071	(0.187)
SECS Social (Pre)	-0.009	(0.015)
SECS Economic (Pre)	0.018	(0.014)
School Education	0.495	(0.492)
Shock Intensity	-0.067	(0.065)
Trait Anxiety	0.034	(0.369)
Political Knowledge	0.090	(0.301)
Political Interest	-0.101	(0.331)
Constant	-3.026	(2.830)
Observations	75	
Log Likelihood	-48.875	
Akaike Inf. Crit.	119.750	
$\chi 2$	6.208615	
**p-value**	**0.7974423**	

Table 3: Randomization test: logit on treatment assignment.

1.2. The 12 Item Social and Economic Conservatism Scale (SECS)

"Please indicate the extent to which you feel positive or negative towards each issue. Scores of 0 indicate greater negativity, and scores of 100 indicate greater positivity. Scores of 50 indicate that you feel neutral about the issue."

- Abortion (reverse scoring). (S)
- Limited government. (E)
- Military and national security. (S)
- Religion. (S)
- Welfare benefits (reverse scored). (E)
- Gun ownership. (E)
- Traditional marriage. (S)
- Traditional values. (S)
- Fiscal responsibility. (E)
- Business. (E)
- The family unit. (S)
- Patriotism. (S)

1.3. Concepts for the Single Target Implicit Association Test (ST-IAT)

1.3.1. Attribute Concept "Positive"

- Joy
- Present
- Love
- Paradise
- Vacation
- Health
- Laughter

1.3.2. Attribute Concept "Negative"

- Stink
- Poison
- Catastrophe
- Disease
- Death
- Pain

1.3.3. Target Concept "Social Conservatism"

- Military and national security
- Religion
- Traditional marriage
- Traditional values
- The family unit
- Patriotism

1.3.4. Target Concept "Right"

- Middle Classes
- Capitalism
- National Flag
- Austerity
- Tradition
- Business Venture
- Economy

1.3.5. Target Concept "Economic Conservatism"

- Limited government
- Fiscal responsibility
- Business
- Gun ownership

1.4. Descriptive Overview of Premeasurements

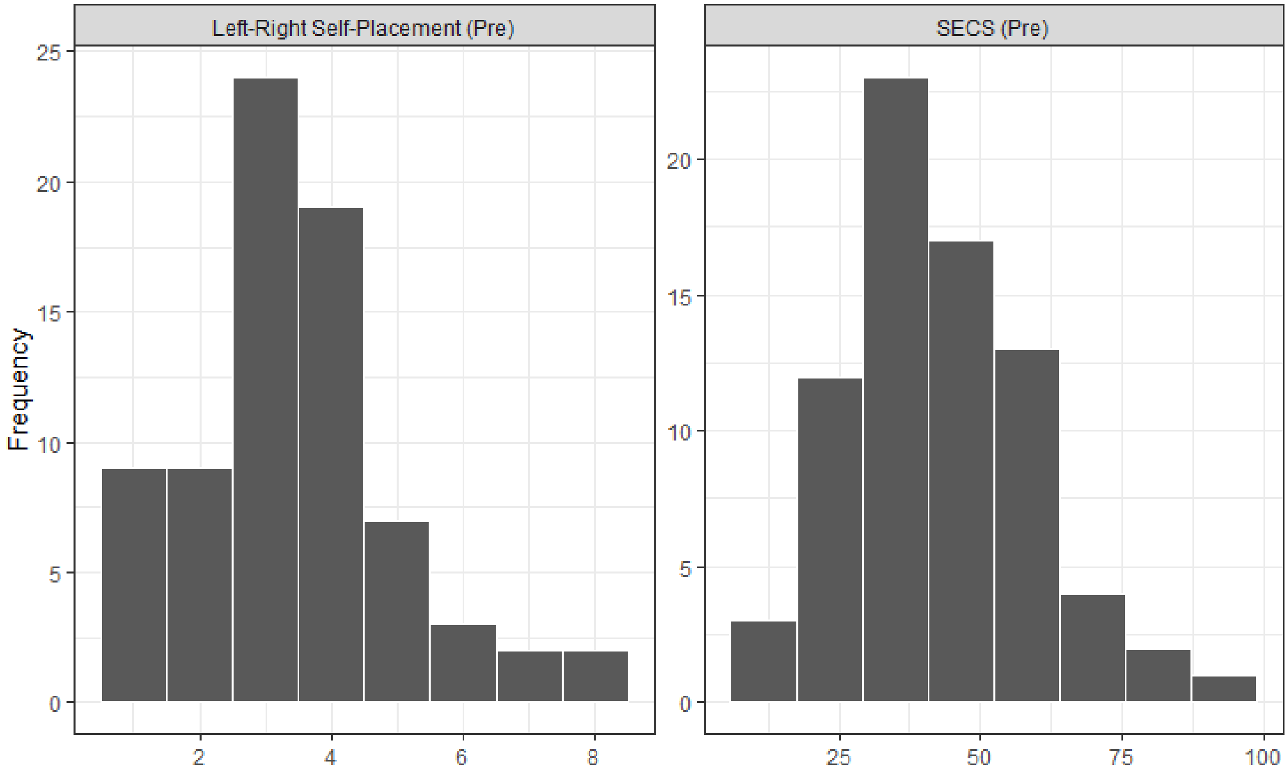

Figure 7: Frequencies of the political attitudes measured in the screening prior to the experiment.

1.5. Heart Rate Data: t-tests of differences between threat and safety

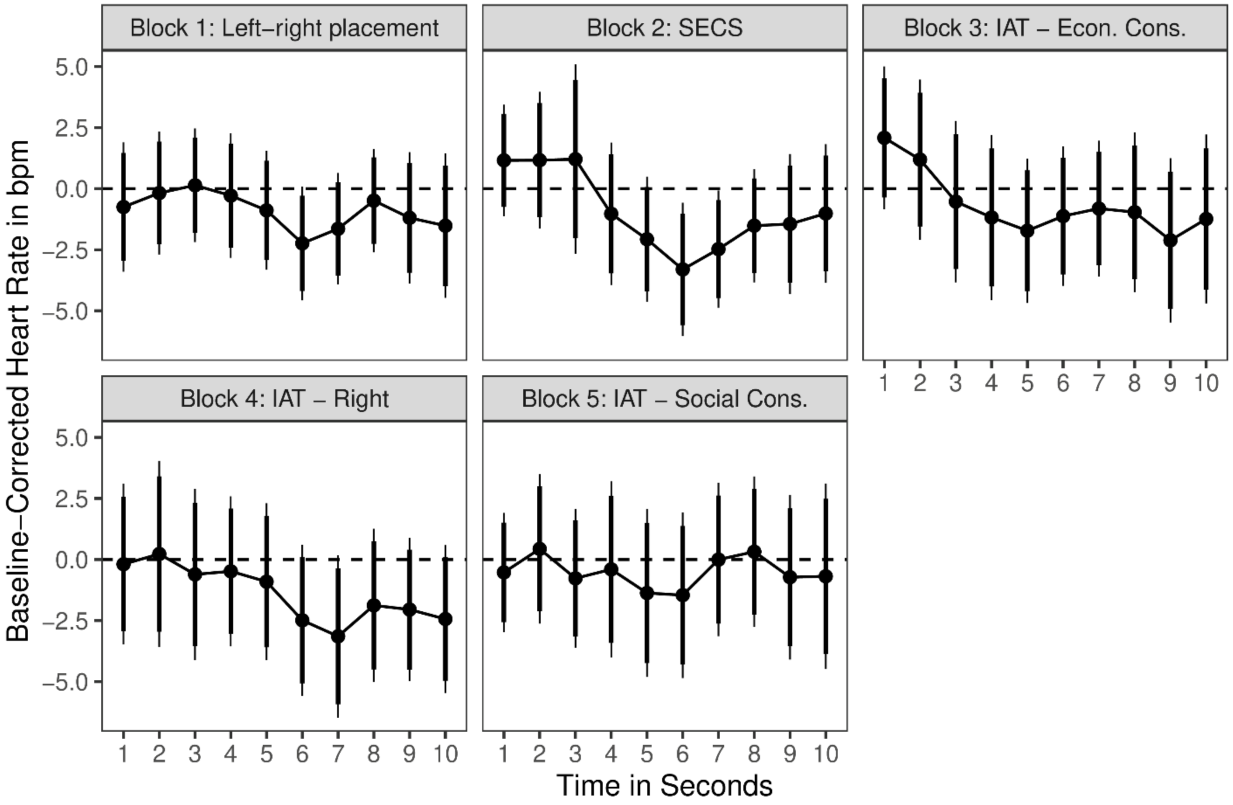

Figure 8: Difference of baseline-corrected heart rates after displaying the signal colour between threat and safety (heart rate under threat – heart rate under safety).

Note: Thin (thick) line indicate 90% (95%) confidence intervals.

1.6. Testing the Conservative Shift Hypothesis: Linear Regression with Control Variables

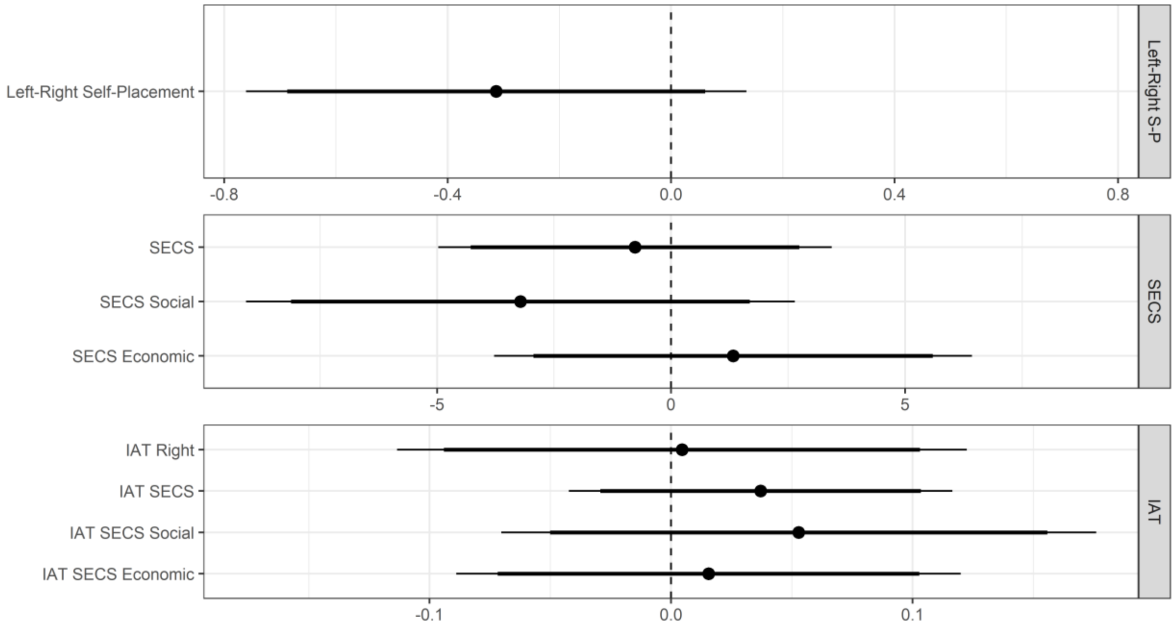

Figure 9: Effect of anxiety on explicit and implicit political attitudes.

Note: N=75. Thin (thick) bars show the 95% (90%) confidence intervals. Effects stem from linear regressions of the outcome variables on experimental condition, prior political attitudes, age, and gender.

1.7. Testing the Conservative Shift Hypothesis: Effects on the single items of the SECS

Instead of only considering effects on the scores of the SECS and the Social Conservatism and Economic Conservatism Subscales of the SECS, we also provide information about how the anxiety manipulation affected the scores of the single items of the SECS. Because the SECS items cover political attitudes towards particular topics and since previous research effects of anxiety sometimes emerged on specific attitudes, e.g., immigration (Brader et al., 2008), we also checked effects on the item-level. Again, *t*-tests focusing on the difference between participants assessed under threat and those assessed under safety consistently show no statistically significant effect on anxiety manipulation. Analogous to above, Figure 10 provides Cohen's *d* as an effect size measure. Again, the point estimates for Cohen's *d* are not consistent in their direction both within the group of items belonging to the Social Conservatism Subscale and those belonging to the Economic Conservatism Subscale.

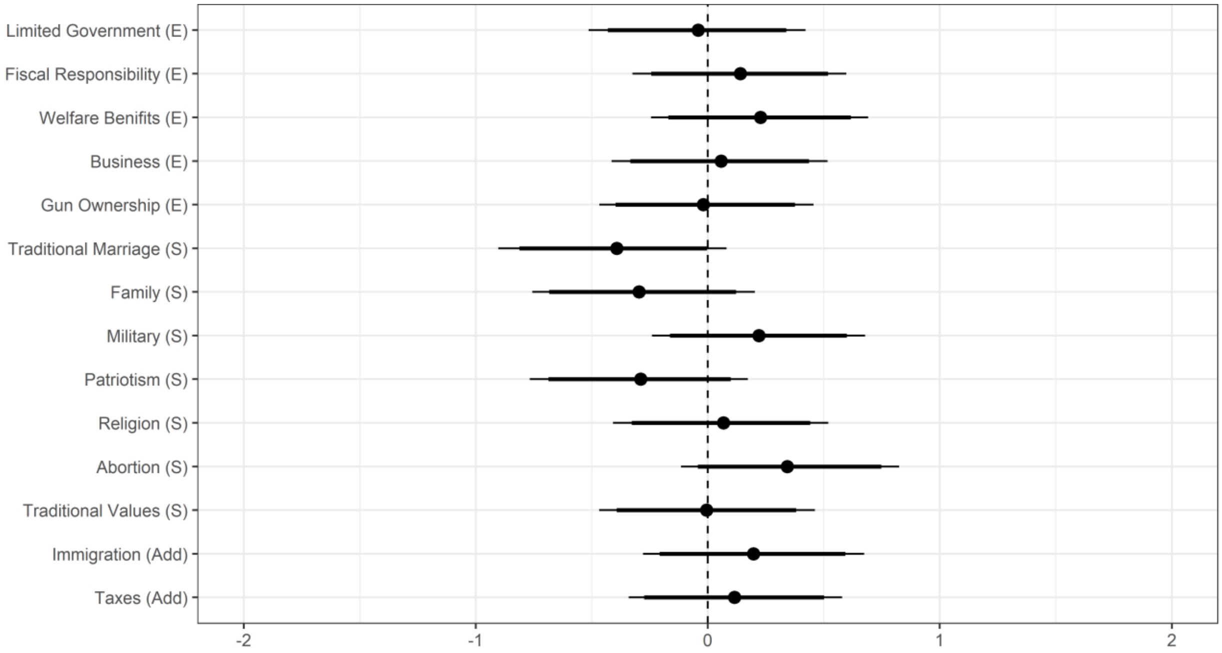

Figure 10: Effect of anxiety on scores on the single items of the SECS expressed as Cohen‘s *d* effect sizes.

Note: N=75. Thin (thick) bars show the 95% (90%) bootstrap BCa confidence intervals. The pooled standard deviation was used for calculating Cohen's *d*. The lowest *p*-value for the SECS item "Traditional Marriage" ($t(73) = 1.70$, $p = .094$).

1.8. Testing the Ideological Intensification Hypothesis: Linear Regression with Control Variables

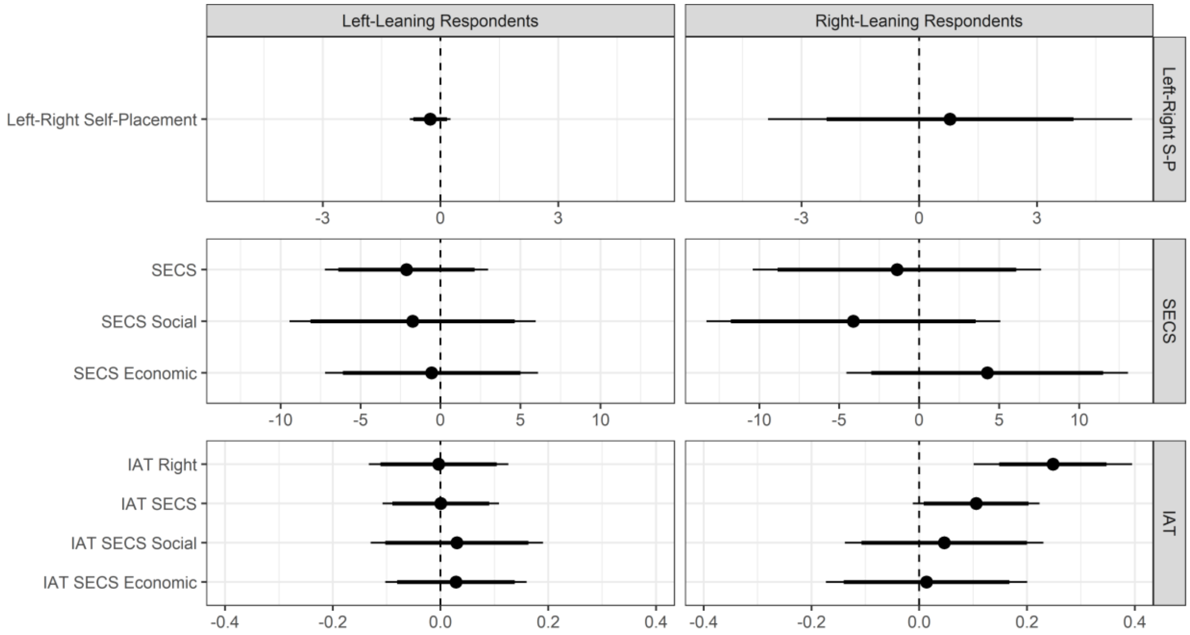

Figure 11: Effects of anxiety on measures of political attitudes in subgroups based on pre-existing political attitudes.

Note: N=75. Thin (thick) bars show the 95% (90%) confidence intervals. Effects stem from linear regressions of the outcome variables on experimental condition, prior political attitudes, age, and gender.

1.9. Testing the Ideological Intensification Hypothesis: Effects on the single items of the SECS

We also did not find significant effects of anxiety on the item-level of the SECS in the subgroups (all $p > .05$). In addition, the effects of anxiety on the item-level in the subgroups did not reveal a consistent pattern in respect to their direction (see Figure 12). In sum, we did not find support for the ideological intensification hypothesis, neither in the explicit nor in the implicit measures of political attitudes.

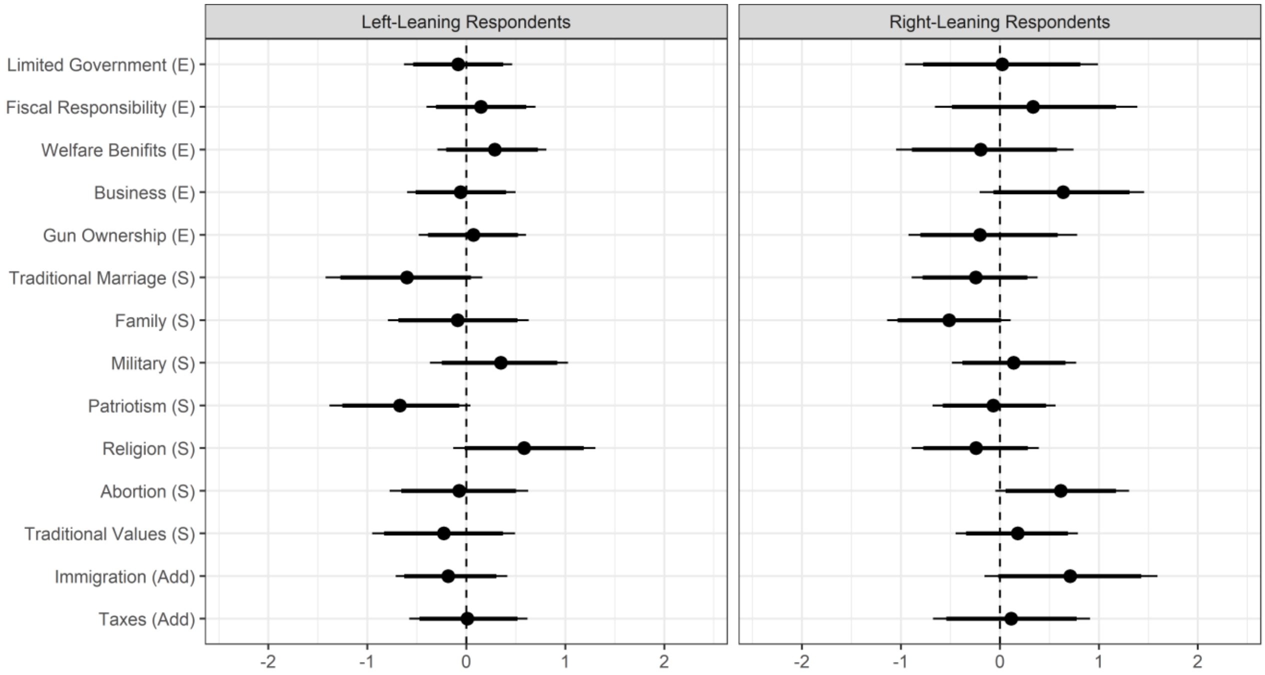

Figure 12: Effects of anxiety on items of the SECS in subgroups based on pre-existing political attitudes.

Note: The dots display effect sizes in Cohen's *d*. Thin (thick) bars show the 95% (90%) bootstrap BCa confidence intervals. The pooled standard deviation was used for the calculation. N varies per subgroup and measure because corresponding pre-measures were used to define subgroups. See data analyses (Left Panel: Economic Subscale (E): $N = 54$. Social Subscale (S): N = 33. Right Panel: Economic Subscale (E): $N = 7$. Social Subscale (S): N = 42)

1.10. Testing the Ideological Intensification Hypothesis: Folding Scales

In an additional analysis, we do not separately assess the ideological intensification hypothesis for participants on the left and right. Instead, we "fold" the scales at the midpoint, which is 5 (50,0) for the Left-Right Self-Placement scale (for the SECS scales, for the IAT scales). Hence values on the folded scales indicate the absolute distance of the values on the old scales to the midpoints of the old scales. These folded scales allow us to assess whether or manipulation induced changes in attitudinal extremity.

t-tests show that also this procedure does not provide evidence for the ideological intensification hypothesis. The manipulation did not induce more extreme values on the folded Left-Right Self-Placement, $t(73) = 1.46$, $p = .148$, the folded SECS score, $t(73) = 0.45$, $p = .651$, or the folded Economic Conservatism Subscale of the SECS, $t(73) = 0.32$, $p = .749$. For the folded Social Conservatism Subscale of the SECS we find that participants assessed under threat display significantly less extreme values on the scale ($M = 8.82$, $SD = 8.08$) than participants assessed under threat ($M = 14.39$, $SD = 12.35$), $t(73) = 2.30$, $p = .024$. This is not consistent with the hypothesis of ideological intensification, but in direct contradiction to it. Also, the participants assessed under threat did not score differently than participants assessed under safety on the folded IAT measures for the concepts “Right”, $t(73) = 0.37$, $p = .71$, “Social and Economic Conservatism”, $t(73) = 0.49$, $p = .629$, “Social Conservatism”, $t(73) = 0.32$, $p = .750$, or “Economic Conservatism”, $t(73) = 0.11$, $p = .913$. In sum, there is no evidence for the ideological intensification hypothesis in this alternative analysis.

In addition, we also repeat the Bayesian analyses using the folded scales. For the ideological intensification hypothesis, the the rival hypotheses on the folded scales are $H_0 : \delta = 0$ and $H_1 : \delta > 0$, where δ is defined as the effect size of the manipulation on our measures. Again, as prior distribution for the alternative hypothesis, we selected a Cauchy distribution with location of 0 and scale of $1/\sqrt{2}$.

The Bayes factors for Bayesian independent samples t-test with the prior distribution defined above are shown in Table 4. All Bayes factors are greater than 3, indicating substantive support for the null hypothesis.

	**Ideological Intensification Hypothesis**
	All Participants
Left-Right Self-Placement	9.39
SECS	5.68
SECS Social	12.68
SECS Economic	3.25
IAT Right	5.40
IAT SECS	5.79
IAT SECS Social	5.22
IAT SECS Economic	4.53

Table 4: Bayes factors in the direction of the null hypothesis for the ideological intensification hypothesis for the folded scales.
